# Revisiting public health programming in Nigeria: challenges and solutions

**DOI:** 10.2147/IJGM.S203172

**Published:** 2019-05-02

**Authors:** Obinna Ositadimma Oleribe, Okey Nwanyanwu, Eric Yi-Liang Shen, Simon D Taylor-Robinson

**Affiliations:** 1 Excellence and Friends Management Care Centre (EFMC), Abuja, Nigeria; 2 Global Health Services Network, Farmington, MI, USA; 3 Department of Radiation Oncology, Chang Gung Memorial Hospital and Chang Gung University, Taoyuan 333, Taiwan; 4 Faculty of Medicine, Imperial College London, London W2 1PG, UK

**Keywords:** programming, supervisory site visits (SSVs), capacity development

## Abstract

Public health programming has three main components – capacity development, service provision and documentation with monitoring. However, most funders and programmers now focus on just documentation and monitoring. In this communication, the authors extensively discuss the need for the full complement of public health programming and why it is important to restructure supportive site visits to make them both empowering and impactful to the health care workers resulting in higher quality of public health services and documentation with monitoring. The authors are of the view that following problem identification, comprehensive capacity development of field workers will engender quality service provision and appropriate documentation and monitoring.

## Introduction

Nigeria is the most populous country in Africa. Although a Federation of different states, it is complex to govern and has suffered from decades of mismanagement over the years and a deeply-scarring civil war that cost many lives, the social effects of which are still felt today in surviving generations. Federal government-driven and non-governmental organization (NGO)-operated funded programs are common in Nigeria and have supported several public health interventions in the country in later years. To ensure effective and efficient use of resources, and accurate reporting to funders, supportive supervisory visits (SSVs) involving funders, government and implementing partners are commonly used. Public health programming has three main components: Capacity development, Service provision and Documentation with monitoring (CSD). Public health programs succeed and survive when practitioners and organizations use inno vative strategies, high impact evidence-based interventions and effective performance management. This is bolstered by effective partnerships and collaborations with public and private-sector organizations. Communication of accurate and timely information to stakeholders is a key and political commitment to obtain resources that support effective and results-oriented action(s) is vital.^1^

All that seems to have been done in Nigeria over the past 10 years is merely program and disease monitoring. This is public health dysfunction of the highest form. In this article, we aim to identify the processes and components of public health programming in Nigeria today to highlight program areas in need of improvement and to ensure a holistic approach to change with a view to better outcomes and impact.

Our literature search was performed through PubMed with the following MeSH terms: Nigeria, public health programming, capacity development and supervisory site visits.

## Problem statement

All public health programs begin with the identification of key problems.^2^ Most of the time, this will be an agency-identified problem. In a few cases, it is driven by the perceived needs of the people served, but there are always issues of great import like the HIV epidemic, Lassa fever outbreaks, malnutrition, lack of access to good water supplies, the absence of healthy, effective and sanitary toilets, rising poverty, increasing cancer prevalence, damage from seasonal flooding, increased school dropout rates, malaria mortality and morbidity, the threat of continued polio outbreaks and its consequences, and maternal deaths from preventable causes.^3^ Other problems can of course be added to this list at great length. The key is that there should always be an identified problem at the root of every public health intervention.

Funders may have additional non-public health agendas, but the primary business case in most programs is usually a public health issue of pressing need. Once this problem is identified (by the agency, funder, government or the people), public health programming is galvanized to minimize/reduce, or completely end the negative impact of these perceived threats, such as HIV and AIDS.^4^

In 2006, one of the authors of this paper joined the group of physicians working to end, or at least ameliorate the traumatic consequences of HIV and AIDS in the world. To do this, he joined the US Center for Diseases Control and Prevention in Nigeria as a Program Specialist, and later went to Tanzania as a Resident Advisor.^5^ When he returned to Nigeria in 2010, he continued his work with NGOs, funders and the government to not only tackle the significant legacy of HIV and AIDS, but also other public health concerns like malaria, hepatitis B, tuberculosis and several other diseases.^6^ He also got involved in other aspects of public health work, such as capacity development, system strengthening and operational research. More than 10 years later, it has become increasing clear to him and his colleagues that public health programming, especially in Nigeria, is completely not effectively managed.

Today, the emphasis is different, being focused primarily on targets and data monitoring. Nothing significant is done about other vital components of public health programming.^7^ As program management professionals, we must try to remind ourselves of what is meaningful in public health programming.

## Public health practice as at today

The majority of funders, organizations and people currently working in the public health eco-space focus on numbers. This is good, but when these numbers are scrutinized just as numbers, and the underlying fact is forgotten that each data point represents a client, or a person with a family, loved ones, relationships and visions, crucial issues are missed.

Staff of funding organizations visit supported sites on at key times just to collect data on numbers enrolled. We know that evidence is central to public health work;^8^ and it is important to public health practitioners and their partners. It is also paramount to policy and decision makers at local, state, national, regional and international levels; key non-governmental stakeholders; and researchers on population health issues. When these numbers (evidence) are not there, emotions sour.^8^ In supported facilities and sites, over 90% of visits by program staff are to ensure that work is documented and all registers and documents are duly completed.^9^ When this is not done, program staff take over the completion of these files and registers to ensure that future monitoring visits have positive outcomes.

Funders, government and interest groups periodically visit these public health sites around Nigeria, which are also called services delivery sites. Although the majority of their site visits are termed SSVs, the visitors appear at sites primarily to collect data, review registers, audit data and score the sites, based on routine documentation.^3^ They visit the sites as monitors or issue identifiers with checklists in hand to see what is working and document it, and at the end of the visit, develop a long report on what should have been done, but was never done. This is important, but is this comprehensive enough? We do not think so, for the reasons outlined below.

Today, rather than site staff looking forward to SSVs from funders, government officials and NGOs across Nigeria, they find these visits an inconvenience, as they see them as distractions and unnecessary. One will wonder why this is the case. Site staff are largely angry and dissatisfied, because these visits do not add any value to their lives, careers, capacity and finances. Rather, such visits mostly waste time that could have been invested in seeing and helping patients. Site staff are not happy that each set of visitors only come to collect information – to take from them – and not to give or add value to their work. Site staff generally view the process as empty “box-ticking”, without any educational value or mentorship.

Rather than waiting with excitement and expectations for SSVs, sites proactively prepare for these visits, making the visits more important than the programs themselves. These site preparations include making sure that all program documents are completed and up-to-date, as this is what guarantees positive scores in the various checklists. Emphasis is never placed on the other components of public health programming, such as patient welfare, staff capacity and quality of service delivery.^10^ Whenever quality is mentioned, it is with respect to the data collected and analyzed and not the quality of services rendered or satisfaction levels of the patients. There is a need to quickly return to convention in the tradition of comprehensive public health programming. Once a problem is identified and funding is secured, the first step in public health programming is capacity development.^4^

## Capacity development

Although this is the first step, it is also an ongoing process that continues all through the implementation period of the program. Site staff are first informed of the need to implement the program to tackle the identified problem(s). With their full buy-in, personnel are trained in the various fundamentals, requirements and strategies for the implementation of a program. This is not with respect to staff orientation, but rather, comprehensive staff training. This training covers the basic cause of the problem(s) identified, incidence and prevalence, the factors that sustain and facilitate them, the identified strategies to resolve the problem and the best ways to document the services rendered.^11^ This is the C of CSD. In most public health programming today, this is largely absent. Where there is any form of training or orientation, it is geared toward better documentation and reporting. We do not contend that documentation and reporting are wrong or not important, but that what is being documented and reported is ineffective, as it is only the bureaucratic part of the process without the educational and nurturing aspects which are required for effective implementation.

Given that capacity development is critical, this continues all through the life cycle of a project, as periodic in-service training, on-the-job mentoring, SSVs and program reviews and retreats. However, because most visits are data related, little is done during these visits to build the capacity of health care workers. We believe that it is wrong to expect untrained staff to deliver high-quality services in a field in which they are ignorant.^12^ Furthermore, in most of these monitoring visits, the well-being of the clients or patients is never discussed, but just the completeness of the data: meeting reports, scorecards, registers, folders and so on. Even when these datasets are corrupted or adulterated, many care little about the purity and sanctity of the process.

In public health programming, site staff should be excited to host visitors if the visitors are coming to add value to their work, life and careers. Site visits should be opportunities to share with workers the relevant skill sets needed to deliver high-quality services. The focus of supervisory visits should not just be to identify what is not working and write reports on them. They should, at least, include on-the-job mentoring, training and support to staff to deliver their work packages. Visitors should plan to spend an adequate amount of time in each site with nothing <3 working days to provide support to an entire program. Site visit agendas should be tailored to include general interaction with staff. Insight should be directed at program progress, identification of challenges and problems to effective programming. Mediation planning and its immediate implementation should be focused upon, with the sharing of best practices, as well as on-the-job mentoring of staff on quality care.^4^ These factors cannot be achieved within a few hours that are currently the length of most monitoring visits. There is, therefore, the need to change the current practice of “arrive, talk and collect data”, to one geared toward accommodating the desires and needs of the sites, adding value to their work and lives.

It will not be out of place for site visitors, as part of their mediation plans, to provide needed equipment that will help the program officers deliver better services. They should also work with the site staff to show them how the work should be done. They should take responsibility to train one or more site staff on site, or in their various institutions (within or outside Nigeria), to ensure they have the right set of skills to deliver quality services.^13^ It is important to understand that just establishing continuous quality improvement teams, SWITCH Teams and Project Management Teams, without empowering them with the right skill sets and tools to deliver quality services, will not achieve the objectives desired.^13^

## Service delivery

The second component of public health programming is service delivery.^14^ The main reason for building the capacity of the health care workers is to deliver services. Once properly trained, they should be equipped to put their new sets of knowledge and skills into ameliorating the sufferings of people or solving the identified public health challenge at hand, such as malnutrition, HIV/AIDS and gender-based violence.

It is very instructive to note that training without the right set of tools and equipment will not suffice. One must ensure that people are given what they need to deliver programs effectively and efficiently. Once staff are trained and equipped to provide services, visits by a supportive supervisory team should be geared toward ensuring that they are doing what they were trained to do and are using the equipment and tools appropriately. This can be achieved when the visitors work with the trainees and mentor them on the job. This cannot be done in a few hours, as is the case currently.

Once staff are well trained in all areas of public health programming, their documentation, and report writing/submission will automatically improve. In a situation where a partner is funded to provide services, while another is to provide training of staff, there will usually be discordance of due process. Challenges may arise when the training partner’s schedule may not align with that of the service provider, and/or the trainers' target audience may be in variance to the service provider’s needs. There is the need to harmonize this.^4^

## Documentation with monitoring

Documentation with monitoring is the last component of public health programming and is critical to ensuring that the right things are done, the right results are obtained and the right structures are ultimately established.^15^ Documentation with monitoring should be a way of providing proof to the funders and to the world that the right investments were made and appropriate results, outcomes and benefits have been obtained.^7^ Documentation should be done, because a programme that is not documented is a programme that never happened.^16^,^17^ Properly trained staff will need no additional encouragement to properly document their work and output. However, because collectively, we are not doing what we should be doing in Nigeria, as far as training is concerned, over 95% of visits to program sites today focus just on data – data entry, collection, collation and also data cleaning, without addressing the wider picture.^18^ This is clearly suboptimal and leads to further disillusionment and ineffectiveness.

When funders ask for a sustainability plan, we wonder what they mean, because from all indications, nothing is ever put in place to ensure the ultimate sustainability of the funded projects. Workers are not properly trained; supervisory visits are simply issue identification visits; mentoring on quality service provision is not taking place; funds are not earmarked to seek and leverage private–public investments; and trained or oriented health care workers are not equipped with the right tools to deliver the right quality of services.

## Conclusion

It is time to return to full public health programming: first, capacity development, then service delivery and finally, documentation with monitoring, in that order. If every practitioner subscribes to the concepts shared here, public health will enjoy better health, and financial returns on tax payers–payers’ investments.^8^

This article is not undermining the importance of monitoring site visits, as these evaluations have been shown to improve the outcome of projects and programs;^19^,^20^ however, we believe that restructuring these monitoring exercises will make public health programs more productive, sustainable and empowering. Studies further suggest that formal mentoring can improve some elements of the work without necessarily improving the general outcome, at least in the short term.^20^

To achieve the objectives of well-planned supervisory site visit, the Capacity Development Results Framework (CDRF or the Framework), which is a powerful approach to the design, implementation, monitoring, management and evaluation of development programs can be used.^21^ This Framework can be used to monitor projects during implementation with a view to taking corrective action. It can also be used to assess the design and results of completed projects or serve as a step-by-step guide to the planning, implementation, and evaluation of projects and programs.^21^ We believe that to maintain workers satisfaction, we must go beyond data collection to capacity development and support for quality service delivery.^22^,^23^

Documentation with monitoring, which is a key component of recent SSVs in Nigeria, is not being conducted effectively and the approach should be modified. SSVs should be long enough to provide training, mentoring and “on-the-job” monitoring for program activities.^24^

To achieve this, it is the responsibility of funders to insist on more comprehensive public health program implementation; the international community to ensure that quality services, not just quality data, are at the core of every funded project; government at state or federal level to ensure that every Memorandum of Understanding or Letter of Agreement signed has adequate provision in the scope of work or terms of reference for staff capacity development, quality service delivery and effective documentation with monitoring components.^25^,^26^

All stakeholders should ensure that every partner plays its part toward sustainable public health service provision. All partners must work together for speedy and effective implementation.

## What is already known about this topic?

1. Funded programs are common in Nigeria and have supported several public health interventions in the country.
2. SSVs are major avenues of monitoring the effectiveness and efficiency of services and funds donated.

## What this paper adds

1. Documentation with monitoring, which is the key component of recent SSVs in Nigeria, is not being conducted effectively and the approach should be modified.
2. Funded programs should include extensive capacity development, service delivery and documentation with monitoring components as the CSD of programming.
3. SSVs should be long enough to provide training, mentoring and “on-the-job” monitoring for program activities.
